# Is Penicillin Allergy a Risk Factor for Early Dental Implant Failure? A Systematic Review

**DOI:** 10.3390/antibiotics10101227

**Published:** 2021-10-09

**Authors:** Angel-Orión Salgado-Peralvo, Juan-Francisco Peña-Cardelles, Naresh Kewalramani, Iván Ortiz-García, Álvaro Jiménez-Guerra, Andrea Uribarri, Eugenio Velasco-Ortega, Jesús Moreno-Muñoz, Enrique Núñez-Márquez, Loreto Monsalve-Guil

**Affiliations:** 1 Department of Stomatology, University of Seville, 41009 Seville, Spain; ivanortizgarcia1000@hotmail.com (I.O.-G.); alopajanosas@hotmail.com (Á.J.-G.); evelasco@us.es (E.V.-O.); je5us@hotmail.com (J.M.-M.); enrique_aracena@hotmail.com (E.N.-M.); lomonsalve@hotmail.es (L.M.-G.); 2 Science Committee for Antibiotic Research of Spanish Society of Implants (SEI—Sociedad Española de Implantes), 28020 Madrid, Spain; juanfranciscopenacardelles@gmail.com (J.-F.P.-C.); k93.naresh@gmail.com (N.K.); 3 Department of Basic Health Sciences, Rey Juan Carlos University, 28922 Madrid, Spain; auribarride@gmail.com; 4 Department of Nursery and Stomatology, Rey Juan Carlos University, 28922 Madrid, Spain

**Keywords:** antibiotic prophylaxis, preventive antibiotics, clindamycin, penicillin allergy, dental implants, dental implant failure

## Abstract

The prescription of preventive antibiotics in dental implant treatments reduces the incidence of early failures. This study has focused mainly on the influence of amoxicillin, which is contraindicated in penicillin-allergic patients. The present systematic review aimed to determine whether penicillin-allergic patients have a higher risk of implant failure compared to non-allergic patients. An electronic search was performed on Medline and Web of Science using the following MeSH terms: (penicillin allergy OR clindamycin OR erythromycin OR azithromycin OR metronidazole) AND (dental implant OR dental implant failure OR dental implant complications). The criteria employed were those described in the PRISMA^®^ Declaration. Only five articles were included that analyzed the failure rates of implants placed in penicillin-allergic patients who were prescribed clindamycin compared to non-allergic patients who were prescribed amoxicillin. With the limitations of this study, it is not possible to state that penicillin allergy per se constitutes a risk factor for early dental implant failure as most of the studies included self-reported allergic patients. Clindamycin has been associated with a significantly elevated risk of failure and an up to six times increased risk of infection. Immediate implants also have a 5.7 to 10 times higher risk of failure.

## 1. Introduction

Dental implants are currently the most predictable therapeutic option for total or partial replacement of missing teeth, with high survival rates of around 95% according to different studies, both in pristine bone and in regenerated bone [1]. Despite this, some implant failures occur [2]. Chrcanovic et al. [3] defined implant failure as those signs and symptoms that lead to the explantation of the implant, whereby “failure” is equivalent to implant loss. The failure rate has been estimated to be around 0.7–3.8%. These failures are classified as “early” or “late” depending on whether they take place before or after, respectively, the functional loading of the implants with a prosthetic restoration [4]. This differentiation is important because different etiological factors are associated depending on the time of their occurrence. In this regard, early failures are caused by a failure of osseointegration due to local and/or systemic factors and account for approximately 5% of all failures, affecting more women and younger patients [2,5]. In contrast, late failures are usually due to bacterial infections, parafunctional habits or mechanical factors related to the implant-supported prostheses and affect the 95% of implants that reach osseointegration [5].

To avoid early failures, Branemark et al. [6] originally suggested that protocols for implant placement should include the administration of phenoxymethylpenicillin 1 hour before surgery and for 10 days postoperatively. This approach was introduced due to the presence of more than 500–700 bacterial species in the oral cavity, in addition to other non-culturable microorganisms discovered by molecular biological techniques [7,8] that may contribute to the development of postoperative infections. Therefore, antibiotic therapy in oral implantology can be classified as either prophylactic/preventive (to prevent infections) or therapeutic (as a treatment for infections already established) [9]. Dentists are often faced with the dilemma of whether or not to prescribe antibiotics preventively in dental implant treatments, and this is currently a controversial issue. The prescription has been accepted not only to avoid systemic bacteremia [10] but also to achieve an adequate antibiotic concentration in the blood in order to prevent bacterial contamination during the surgical placement of implants or grafted material [11]. Amoxicillin is the most studied antibiotic for this purpose; however, antibiotics other than beta-lactams in penicillin-allergic patients are not sufficiently studied.

The most common adverse drug reactions associated (≥1% of patients) with penicillin use are diarrhea, nausea, rash, neurotoxicity, urticaria and/or superinfection (including candidiasis). Infrequent adverse effects (0.1 to 1% of patients) are fever, vomiting, erythema, dermatitis, angioedema, seizures (especially in epileptics) and/or pseudomembranous colitis. Despite this, only 0.01% of patients treated with penicillin have their lives compromised by experiencing true anaphylaxis, i.e., hypersensitivity with hypotension, angioedema, bronchospasm and urticaria [12]. Currently, some organizations such as the American Heart Association [13] (AHA) have stopped recommending clindamycin as an alternative antibiotic for antibiotic prophylaxis against infective endocarditis.

The present systematic review aimed to determine whether penicillin-allergic patients have a higher risk of implant failure compared to non-allergic patients.

## 2. Materials and Methods

### 2.1. Search Strategy

A search was performed in the Medline/PubMed database and through the Web of Science (WoS) with the following MeSH (Medical Subjects Headings) terms: (penicillin allergy OR clindamycin OR erythromycin OR azithromycin OR metronidazole) AND (dental implant OR dental implant failure OR dental implant complications). In addition, we searched Google Scholar for articles that met the criteria described above and examined the bibliographic references of the selected articles for publications that did not appear in the initial search and might be of interest. The search was not time-restricted and was updated to March 2021.

The criteria used were those described in the PRISMA^®^ (Preferred Reporting Items for Systematic Reviews and Meta-analysis) Statement [14]. The present systematic review aimed to answer the following “PICO” question (P = patient/population/problem; I = intervention; C = comparison; O = outcome) (Table 1):

In penicillin-allergic patients who have not been prescribed these antibiotics, is there an increased risk of dental implant failure compared to non-allergic patients?

In case the article did not specify whether the penicillin allergy was diagnosed with a specific test beforehand or whether it was declared by the patient, the authors contacted the corresponding author for clarification.

### 2.2. Eligibility Criteria

Before starting, exclusion and inclusion criteria were defined for the resulting articles.

#### 2.2.1. Exclusion Criteria

The exclusion criteria determined the exclusion of the following: (a) experimental laboratory studies; (b) animal studies; (c) studies whose main topic was not the analysis of early dental implant failure in penicillin-allergic patients; (d) duplicate articles; (e) books or book chapters; (f) letters to the Editor; and (g) commentaries.

#### 2.2.2. Inclusion Criteria

Included studies were: (a) human studies; (b) articles published in English or Spanish; (c) meta-analyses; (d) systematic reviews; (e) randomized clinical trials (RCTs); (f) cohort studies; (g) observational studies; (h) comparative studies; and (i) multi-centric studies.

### 2.3. Study Records

Two researchers (A.-O.S.-P. and J.-F.P.-C.) independently compared the results to ensure completeness and removed duplicates. Then, the full title and abstracts of the remaining papers were screened individually. Finally, full-text articles to be included in this systematic review were selected according to the criteria described above. Disagreements over eligible studies to be included were discussed with a third reviewer (N.K.) and a consensus was reached.

### 2.4. Risk of Bias

Data collection was conducted using a predetermined table designed in advance of the assessment of the resulting articles. Two independent reviewers (A.-O.S.-P. and J.-F.P.-C.) evaluated the methodological quality of eligible studies following the Joanna Briggs Institute (JBI) Critical Appraisal Tool [15], which incorporates 10 domains. The studies were classified as low-quality assessment studies (0–5 domains), or as high-quality assessment studies (5–10 domains).

## 3. Results

### 3.1. Study Selection

**Figure 1:**
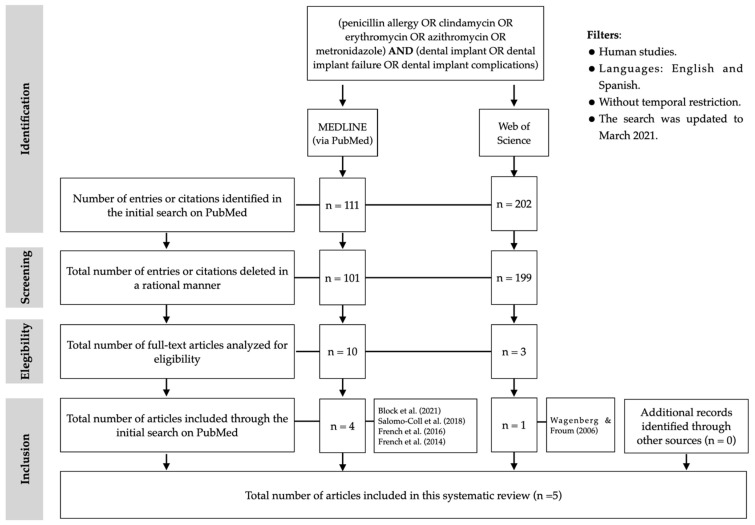
PRISMA^®^ flow diagram of the search processes and results.

After the initial search in Medline/PubMed, 111 articles were found. After reading the titles and abstracts, 101 articles were excluded because they either did not include penicillin-allergic patients or were not related to the purpose of the study. Therefore, 10 full-text articles were considered, of which 6 were excluded because they did not analyze the failure rates of dental implants in penicillin-allergic patients. Finally, four articles were included through the PubMed search. Through WoS, after analyzing the 202 titles and abstracts and eliminating duplicate articles, 3 full-text articles were evaluated, of which only 1 met the criteria described. No articles were identified and included through other channels. Therefore, five studies were included in the present systematic review (Figure 1 and Table 2). Four were observational cohort studies [16,17,18,19] and one was a retrospective case-control study [20]. 

### 3.2. Study Characteristics

The included studies only evaluated the effect of clindamycin as an alternative antibiotic in antibiotic prophylaxis in penicillin-allergic patients, in the placement of implants in native bone with or without the need for simultaneous guided bone regeneration (GBR) and/or sinus lifts and immediate implants. Of the five studies, allergy testing to confirm penicillin allergy was performed in only one study [19]. The remaining four studies included patients with self-reported penicillin allergy (SRPA) [16,17,18,20]. The main findings of each investigation are described below.

Salomó-Coll et al. [16] (2018) described failure rates in patients non-allergic to penicillin of 8.03%, while in the group of patients with SRPA the failure rates were 24.68%, i.e., one in four implants failed (*p* = 0.032), with a relative risk (RR) of 3.84. Clindamycin was prescribed in 100% of these patients. In patients with SRPA, 21.05% of implants failed late, while 78.95% failed early. The reason for early failure was either a failure of the osseointegration process (80%) or uncontrolled infection (20%). At an individual patient level, failure rates were 5.17% in non-allergic patients and 18.86% in patients with SRPA (*p* = 0.046) (RR = 3.64).

French et al. [19] (2015) found twice the risk of implant failure in confirmed penicillin-allergic patients who were prescribed clindamycin versus those who were prescribed amoxicillin (hazard ratio (HR) = 2.16).

However, these results were not significant (*p* = 0.11) due to the low number of allergic patients included and the low failure rates experienced in the whole sample (0.70%). These authors suggest avoiding immediate implant placement in patients in whom penicillin cannot be administered and placing implants on a delayed basis.

French et al. [17] (2016) conducted a similar study in which they described failure rates of implants placed in non-allergic patients of 0.80% (of these, 53.80% were early failures) versus 2.10% in patients with SRPA (80% failed early), these differences being significant (*p* = 0.002), with an odds ratio (OR) of 3.10. Differences in survival rates measured at 1, 5 and 10 years were also significant (*p* < 0.002), being 99.50%, 98.90% and 98.40% in non-allergic patients and 98.10%, 97.30% and 97.30% in patients with SRPA, respectively. These authors also studied the occurrence of postoperative infections, which was 0.60% in non-allergic patients and 3.40% in patients with SRPA, i.e., the risk in allergic patients who were prescribed clindamycin was six times higher (*p* < 0.05). In this study, 12.30% of implants were immediate implants (n = 687), of which 91.7% (n = 630) were placed in non-allergic patients with a failure rate of 1%, while 8.30% were placed in patients with SRPA with a failure rate of 10.50%, which is 10 times higher (*p* < 0.001). These authors relate these differences between the two groups to a higher infection rate in patients with SRPA.

Wagenberg and Froum [18] (2006) carried out an investigation similar to the two studies mentioned above in which they described a 5.70 times higher risk of immediate implant failure secondary to infection in patients with SRPA who were prescribed clindamycin (8.52%) compared to non-allergic patients who were administered amoxicillin (2.95%); these differences were significant (*p* < 0.001) (RR = 3.34).

On the other hand, Block et al. [20] (2021) conducted a retrospective case-control study on 224 patients who experienced one or more implant failures. The logistic regression model found a significant association between implant failures one year after placement in patients with SRPA (OR = 2.98), but not between the first and fourth year, nor after 4 years. These authors did not specify whether preventive antibiotics were administered and, if so, which ones.

### 3.3. Risk of Bias within Studies

Risk of bias and study quality analyses were performed independently by two review authors (A.-O.S.-P. and J.-F.P.-C.). Using the predetermined 10 domains for the methodological quality assessment according to the JBI Prevalence Critical Appraisal Tool [15], it was determined that four of the papers included had a high-quality assessment (5–10 domains) [16,17,19,20] and one of them had a low-quality assessment (0–5 domains) [18]. Table 3 shows a more detailed description of the articles included.

## 4. Discussion

Approximately 10–20% of patients report an allergy or reaction to penicillin; however, these are rarely hypersensitivity or immunoglobulin-E-mediated reactions, so these drugs could be administered safely [21,22,23]. Moreover, studies have shown that 80–99% of patients may no longer be considered allergic after allergy testing [24,25,26].

Several studies have investigated the influence of the type of antibiotic in patients who self-reported allergy to penicillin in various implant procedures [27,28,29,30,31]. In this regard, Khoury et al. [27] (2018) prescribed clindamycin 600 mg 1 h preoperatively followed by 300 mg/8 h/7 days postoperatively in patients with SRPA, while in the non-allergic group they prescribed amoxicillin 2 g preoperatively for antibiotic prophylaxis followed by 10 days of antibiotics postoperatively in sinus lifts with a lateral window approach and a one- or two-stage implant placement. Subantral graft infection occurred in 0.48% of all patients, all of whom were patients with SRPA, which accounted for 6% of all these patients. The infection occurred in the subantral graft and the symptomatology started at 4–8 weeks. None of the patients had a history of sinusitis and there were no surgical complications such as sinus membrane perforation, mucosal dehiscence, graft exposure and/or tissue necrosis.

Basma et al. [31] (2021) studied the incidence of infectious complications on 2,530 socket grafting (SG) and 341 ridge augmentation (RA) procedures performed on 1,814 patients who were prescribed amoxicillin 2 g 1 h before surgery followed by a dose of 500 mg/8 h 7 days postoperatively and, in patients with SRPA, clindamycin 600 mg 1 h before followed by 300 mg/12 h 7 days postoperatively. The results showed postoperative infection rates after SG of 10.7% in the clindamycin group vs. 2.7% in the amoxicillin group (OR = 4.5; *p* < 0.02) and in RA of 22.5% vs. 4.2%, respectively (OR = 6.9; *p* < 0.01). Therefore, the described risk of infection in these regenerative procedures after clindamycin administration is 5.5 times higher compared to amoxicillin (*p* < 0.01).

Two studies evaluated the crestal bone changes after immediate implant placement. Wagenberg et al. [28] (2013) observed that, among patients who were administered penicillin, crestal bone loss was lower (0.52 ± 0.82 mm) compared to the prescription of other antibiotics (0.61 ± 0.86 mm) in patients with SRPA. Although, these differences were not significant. No reference was made to the type of antibiotic prescribed in these patients. The second study conducted by Wagenberg and Froum [29] (2020) had the same objective; however, they compared the administration of amoxicillin 500 mg/6 h one day preoperatively followed by its prescription for 10 days postoperatively, versus azithromycin 250 mg 2 days preoperatively, followed by 6 days postoperatively, with no significant differences at maxillary (*p* = 0.53) or mandibular level (*p* = 0.80). These authors also failed to perform specific tests to confirm an allergy.

Finally, Froum et al. [30] (2018) analyzed the results of peri-implantitis treatment in these patients. They were the only authors to observe an improvement in the parameters studied in patients with SRPA. There were a reduction in probing depth, with values of 5.95 ± 1.72 mm in patients with SRPA vs. 5.52 ± 2 mm in non-allergic patients; radiographic bone gain of 2.30 ± 2.13 mm vs. 1.94 ± 1.76 mm; and soft tissue gain/loss of 0.76 ± 1.46 mm vs. 0.56 ± 1.45 mm, respectively, with these differences not being significant. An important bias of this study is that no reference was made to the type of antibiotic prescribed.

Current evidence has shown that immediate implant placement per se is a risk factor for implant treatment, with early failure rates of 5.1% compared to 1.1% when implants are placed in a delayed procedure, i.e., 6 months after tooth extraction (*p* = 0.02) [32]. Despite this, Wagenberg and Froum [18] and French et al. [17] (2016) found a significantly higher implant failure rate in SRPA patients compared to non-allergic patients, specifically 5.70–10 times higher.

These findings may suggest that clindamycin may be a risk factor for dental implant failure, although further studies are required to confirm this hypothesis. To formulate this hypothesis, literature related to orthopedic surgeries was reviewed, and the same trend was observed. Specifically, recommendations from competent authorities in this field suggest administering 1–3 g of cefazolin 1 h before total knee and hip arthroplasty surgeries. However, due to its beta-lactam ring structure, it is not recommended for administration in penicillin-allergic patients. In these cases, clindamycin or vancomycin is recommended. In a study [33] on 17,026 total knee and 12,669 hip arthroplasties, cefazolin was prescribed in 94.9% of cases and another type of antibiotic in beta-lactam-allergic patients (vancomycin or clindamycin) in the remaining 5.1%, with significantly higher infection rates observed in the latter group. In addition, a higher prevalence of methicillin-resistant *Staphylococcus aureus* (MRSA) was found (17%) compared to the cefazolin group (4.8%) (*p* < 0.001), as well as significantly lower survival rates at years 1, 5 and 10 (*p* = 0.021). Other authors observed a 50% higher risk of MSRA-attributable infection in penicillin-allergic patients who were prescribed other drugs (of these, 49% with clindamycin). According to multivariate analysis, this increased risk was entirely attributable to the prescription of drugs other than penicillin [34]. These findings were corroborated by other authors who observed a significantly higher prevalence of infection in penicillin-allergic patients prescribed vancomycin instead of cefazolin (OR = 1.58; *p* < 0.048) [35], with an increased risk of infection by Gram-negative microorganisms (OR = 2.42; *p* = 0.049) [36].

There are several possible explanations for the presence of an increased risk of implant failure and/or infection in patients who were not prescribed penicillin.

### 4.1. Suboptimal Efficacy of Alternative Antibiotics, Such as Clindamycin

This drug may favor an increase in the proportions of resistant *Prevotella* species in saliva [37] and some, such as *P. intermedia* and *P. aeruginosa*, are often found in implants with peri-implantitis [38]. In this context, an in vitro study found that one or more pathogenic species found in implants with peri-implantitis, especially *P. intermedia*, *Tannerella forsythia* and *Aggregatibacter actinomycetemcomitans*, are resistant at therapeutic concentrations in 46.70% of cases to clindamycin [39]. In addition, several studies have linked the prescription of antibiotics other than beta-lactams to an increase in MRSA [33]. In this regard, *S. aureus* has been found at high concentrations in implants with peri-implantitis, as have other clindamycin-resistant bacteria mentioned above (*A. actinomycetemcomitans*, *P. intermedia* and *T. forsythia*) (*p* < 0.001) [40]. In addition, if *S. aureus* is part of the early colonizing bacteria of implants, this bacterium will be present one year later [41], thus increasing the risk of future peri-implantitis [41,42,43].

On the other hand, preoperative clindamycin treatment and its continuation for 10 days postoperatively may contribute to sinus colonization with clindamycin-resistant organisms [27]. Zirk et al. [44] studied the type of antibiotic appropriate for the treatment of odontogenic maxillary sinusitis, concluding that clindamycin is the antimicrobial with the most unfavorable results, with 50% of tested pathogens resistant [45]. Pigrau et al. [46] (2009) studied the effect of various antibiotics in the treatment of osteomyelitis in a sample in which 92.48% of patients had previously been exposed to clindamycin for various reasons, including 15.22% for prophylaxis before implant placement. These authors observed that *Streptococci viridans* was susceptible in 81% to penicillin and 96% to fluoroquinolones but only 11.5% to clindamycin. At least one clindamycin-resistant species was present in 92.10% of the samples, indicating the rapid emergence of resistance in patients previously exposed to clindamycin. 

### 4.2. Penicillin Allergy as a Genetic Factor Predisposing to an Increased Risk of Implant Failure

Genetic analysis of more than one million people, more than 100,000 of whom had an adverse response to penicillin, identified a genetic variant in the human leukocyte antigen (HLA) genes associated with penicillin allergy. By comparing the frequencies of thousands of polymorphisms between those showing an adverse response to penicillin and those showing a normal response, two regions of the genome related to the former were detected: one located in the HLA-B major histocompatibility complex gene and the other in the PTPN22 gene. The results of the analysis showed that carriers of the HLA-B*55:01 allele have a 33% higher relative risk of penicillin allergy than the rest of the population, which could point to a lymphocyte-mediated predisposition leading to a delayed penicillin reaction [47]. Recent research suggests a link between polymorphisms in these genes and rheumatoid arthritis [48,49], a systemic autoimmune chronic inflammatory disease that has been identified as a risk factor for dental implant failure [50]. Nevertheless, studies are needed to confirm the hypothesis of a possible link between genetic alterations and increased susceptibility to implant failure.

### 4.3. Negative Influence of Clindamycin on Osseointegration

In vitro studies have shown that, at high concentrations, clindamycin reduces the activity of alkaline phosphatase (a marker of osteoblastic metabolism and, therefore, of osteogenic differentiation) and the calcification of the extracellular matrix in a dose-dependent manner, while at low concentrations it increases the metabolism of osteoblasts. It is important to note that the concentrations studied (100–500 µg/ml) are not reached after systemic application but are reached after local administration [51]. Other authors have shown that clindamycin produces cytotoxic and cytostatic effects on primary human osteoblasts due to an impairment of mitochondrial energy [52].

Until more studies are conducted, it is recommended that diagnostic tests are performed to confirm SRPA, and, in positive cases, it seems prudent to avoid the use of clindamycin in favor of other drugs. At present, the evidence suggests that prescribing 2–3 g amoxicillin 1 h before implant surgery in healthy patients in ordinary situations, i.e., without the need for associated regenerative surgery [53], or in bone augmentation procedures, with or without the simultaneous insertion of dental implants [54], is the protocol that has been clinically proven to prevent the most implant failures. For many years, clindamycin was the preventive and therapeutic antibiotic of choice in penicillin-allergic patients, which may have resulted in other types of antibiotics not being extensively studied in our field. For this reason, it is not possible to establish solid evidence-based recommendations in penicillin-allergic patients. Nevertheless, from this systematic review, the authors recommend the use of azithromycin 500 mg 1 h before surgery as an alternative until further studies are conducted. In this sense, compared to the preoperative prescription of 2 g amoxicillin 1 h before surgery, it has shown significant effects on inflammation and early healing, with concentrations of 3.4 (0.7) and 2.8 g/mL (0.9) in gingival and peri-implant crevicular fluid on postoperative day 6, respectively, while amoxicillin concentrations were below detectable limits. Likewise, gingival crevicular fluid levels were significantly lower with azithromycin during the initial healing period. These differences are due to decreased levels of granulocyte colony-stimulating factor (G-CSF), interleukins 6 and 8, macrophage inflammatory protein 1 (MIP-1) and interferon (IFN)-gamma-inducible protein 10 kDa (IP-10), reducing the mobilization of granulocyte precursors and the recruitment of immune and inflammatory cells during the healing phase [52].

This systematic review presents several limitations. First, the low number of studies available in the literature. Second, the heterogeneity of the included studies and the lack of RCTs, so data should be interpreted with caution.

Future lines of research should be directed toward RCTs to study the administration of clindamycin and amoxicillin in patients not allergic to penicillin, as well as the study of the prescription of clindamycin compared to other drugs, such as azithromycin, in patients diagnosed with penicillin allergy through specific tests. Equally interesting would be the investigation of the relationship of polymorphisms associated with penicillin allergy with alterations at the bone level that may have a negative influence on the osseointegration of dental implants.

## 5. Conclusions

It is not possible to state that penicillin allergy per se constitutes a risk factor for early failure of dental implants because most of the studies included patients with SRPA without specific diagnostic tests. The preventive antibiotic used in these patients was clindamycin, showing a significantly high associated risk of implant failure, mainly related to a failure of osseointegration of the implants as well as an increased risk of infection of up to six times compared to other antibiotics. Immediate implants also have a 5.7- to 10-fold increased risk of failure in these patients. Allergy testing is recommended to confirm the allergy, as well as studies aimed at finding an alternative to penicillin in these patients.

## Figures and Tables

**Table 1:** Breakdown of the “PICO” question.

Component	Description
P (problem/population)	DI ^1^ failure
I (intervention)	Prescription of antibiotics other than penicillin
C (comparison)	Comparison between patients with/without allergy to penicillin
O (outcome)	Prevention of DI failure
PICO question	In penicillin-allergic patients who are not prescribed these antibiotics, is there an increased risk of DI failure compared to non-allergic patients?

^1^ DI, dental implant.

**Table 2:** Results of included observational cohort studies.

Author(s)/ Year	Allergy Confirmation	Procedure	N ^1^/DI ^2^		Failure Rates (at Implant Level)				Antibiotics Used	
			NA ^3^	A ^4^	NA	A	RI ^5^	*p*^6^ (95%)	NA	A
Salomó-Coll et al. [16] (2018)	SRPA ^7^	Ordinary implants in healthy patients	1133/2572	77/175	8.03%	24.68%	RR ^8^ 3.84	0.032	Amoxicillin 2g ^9^, 1h PreOp + 750 mg ^10^/8h ^11^/7d ^12^ PostOp	Clindamycin 600 mg, 1h PreOp + 300 mg/6h/7d PostOp
French et al. [17] (2016)	SRPA	Various types: ordinary implants, with simultaneous GBR ^13^, immediate and sinus lifts ASA I or II patients	UNS ^14^/5106	UNS ^14^/470	0.80%	2.10%	OR 3.10	0.002	Amoxicillin 2 g, 1h PreOp + 500 mg at 8h PostOp (If bone-grafting, immediate implant or sinus lift: 250 mg/8h/7d)	Clindamycin 600 mg/1h PreOp (If bone-grafting or immediate implant 150 mg/6h/7d. If sinus lift: levofloxacin 250 mg/12h/7d)
French et al. [19] (2015)	Preliminary test	Placement of implants in native bone with or without GBR, immediate implants and sinus lifts	1898/UNS ^14^	162/UNS ^14^	UNS ^14^	UNS ^14^	HR 2.16	0.11	Amoxicillin (UNS ^14^ guidelines)	Clindamycin (UNS ^14^ guidelines)
Wagenberg and Froum [18] (2006)	SRPA	Placement of immediate implants, some with sinus lift and some with immediate loading	UNS ^14^/1561	UNS ^14^/364	2.95%	8.52%	RR 3.34	<0.001	Amoxicillin 500 mg/6h/2d PreOp + 10d PostOp	Clindamycin 300 mg/6h/2d PreOp + 10d PostOp

^1^ N, sample size; ^2^ DI, dental implants; ^3^ NA, not allergic to penicillin; ^4^ A, allergic to penicillin; ^5^ RI, risk indicator; ^6^ *p*, statistical significance at 95% confidence interval; ^7^ SRPA, self-reported penicillin allergy; ^8^ RR, relative risk; ^9^ g, gram; ^10^ mg, milligrams; ^11^ h, hour(s); ^12^ d, days; ^13^ GBR, guided bone regeneration; ^14^ UNS, unspecified.

**Table 3:** JBI Critical Appraisal Tool for studies reporting prevalence data.

	Block et al. [20] (2021)	Salomó-Coll et al. [16] (2018)	French et al. [17] (2016)	French et al. [19] (2015)	Wagenberg and Froum [18] (2006)
1. Was the sample representative of the target population?	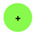	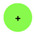	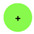	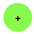	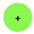
2. Were study participants recruited in an appropriate way?	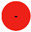	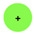	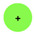	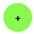	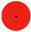
3. Was the sample size adequate?	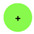	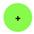	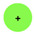	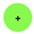	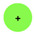
4. Were the study subjects and setting described in detail?	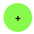	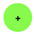	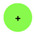	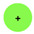	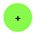
5. Was the data analysis conducted with sufficient coverage of the identified sample?	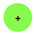	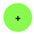	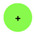	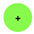	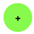
6. Were the objective, standard criteria used for measurement of the condition?	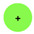	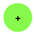	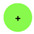	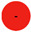	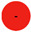
7. Was the condition measured reliably?	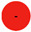	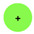	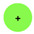	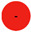	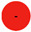
8. Was there appropriate statistical analysis?	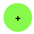	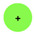	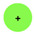	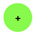	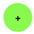
9. Were all the important confounding factors/subgroups/differences identified and accounted for?	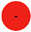	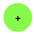	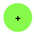	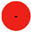	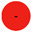
10. Were subpopulations identified using objective criteria?	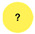	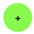	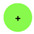	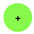	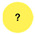

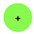
Yes; 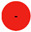
No; 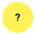
Unclear; 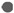
Not applicable.

## Data Availability

Data available in a publicly accessible repository.
